# A Phytobiotic Supersupplement From *Murraya koenigii* Improves Growth, Physiological Health, Skin Colour and Pattern in a Wild Ornamental Fish (*Oreichthys crenuchoides*) Under Captivity

**DOI:** 10.1155/anu/8708940

**Published:** 2025-08-06

**Authors:** Nallaperumal Siva, Gouranga Biswas, Paramita Banerjee Sawant, Sujata Sahoo, Dilip Kumar Singh, Sweta Pradhan, Prem Kumar

**Affiliations:** ^1^Aquaculture Division, ICAR—Central Institute of Fisheries Education, Panch Marg, Off Yari, Versova, Andheri (W), Mumbai 400061, India; ^2^Aquaculture Division, ICAR—Central Institute of Fisheries Education, Kolkata Centre, 32-GN Block, Sector V, Salt Lake City, Kolkata 700091, India; ^3^Fish Nutrition, Biochemistry and Physiology Division, ICAR—Central Institute of Fisheries Education, Kolkata Centre, 32-GN Block, Sector V, Salt Lake City, Kolkata 700091, India; ^4^Fish Nutrition, Biochemistry and Physiology Division, ICAR—Central Institute of Fisheries Education, Panch Marg, Off Yari, Versova, Andheri (W), Mumbai 400061, India

**Keywords:** flavonoids, melanin pattern, *Murraya koenigii*, phytobiotics, wild ornamental fish

## Abstract

Ornamental fish sector is one of the fastest growing industries in the world with an estimated market value of 18–20 billion USD. Despite its global economic significance, it suffers from several issues notably, high-cost carotenoids, inadequate seed production and antimicrobial resistance (AMR). The existence of multifaceted problems needs an integrated multifunctional solution. The phytobiotics of *Murraya koenigii* (PMK), also called as curry leaf is known for its diverse bioactive compounds with rich flavonoids and carotenoids, and can be a silver bullet in dealing with the multidimensional issues in ornamental fish sector. The multifunctionality of the selected phytobiotics was assessed in drapefin barb, *Oreichthys crenuchoides* which is known for its non-chromatic nature with prominent reticulate melanin patterns. The PMK was administered orally at 0, 5, 10, 15 and 20 g/kg diet for 60 days. At the end of the trial, a significant difference was noted in the growth performance and feed utilisation efficiency in PMK-administered groups. The protease and lipase activities were increased significantly by the action of PMK, with the highest enzyme activity recorded at 5 g/kg group and the amylase activity was dropped in all PMK groups, facilitating an improved nutrient assimilation. The PMK regulated liver health, which was evident by the declined levels of alanine aminotransferase (ALT), aspartate aminotransferase (AST) and alkaline phosphatase (ALP) activities in the liver and muscle. Further, haematological profile reflected the enhancement of aerobic metabolism and immunity by PMK. PMK also exhibited antimicrobial activity against selected pathogens. Despite the carotenoid content and mild greenish-yellow tinge on the skin, no significant skin colour changes were observed in chromaticity analysis. Instead, the reticulate patterns in *O. crenuchoides* became prominent due to the rich flavonoid content in PMK. Finally, the performance index and integrated biomarker response (IBR) index pointed out that 10 g/kg PMK as an effective dose of administration to *O. crenuchoides* considering its efficacy.

## 1. Introduction

Ornamental fish sector is one of the fastest-growing industries in the world with an estimated annual market value of 18–20 billion USD and it is constantly expanding [1]. In the twenty-first century, it not only caters to the increasing consumer demand for domestic and public aquaria but also exhibits a crucial role in local and global economies. The ornamental fish trade generates substantial economic benefits including job generation, tourism and contribution to biodiversity conservation efforts [2]. This interplay between economic growth and environmental stewardship positions it as a vital component of contemporary aquaculture and a potential model for sustainable resource management.

The aquariculture sector is principally driven by aesthetic features like size, colour, pattern, etc. which determine the market value of the aquarium fish, as consumers often seek visually striking specimens to enhance their aquatic exhibits. These vibrant colours and unique patterns which are attractive traits of ornamental fish not only attract enthusiasts but also influence breeding operations and market trends [3]. Despite its glorifying significance, ornamental fish sector faces several challenges, particularly in affording an economic carotenoid source, seed production and disease management [4, 5]. However, phytotherapeutic approach using diverse metabolites of plants can potentially serve as a silver bullet which resolves multiple problems of the industry in a single integrated approach.

Size is one of the principal determinants of market value in ornamental fish trade [6]. Bigger-sized fishes are often more desirable in aquarium because of their impressive visual displays. Tiny fish are ideal candidates for nano aquarium which is more convenient to handle and doesn't take much space. Slow growth rates in aquarium fishes poses significant challenges to the aquariculturist as the fishes with sluggish growth take long time to develop aesthetic features. The slow growth rate creates an imbalance in the aquarium by pre-mature death of weaker fish and territorial dominance among fishes.

Disease outbreak is another major menace causing significant loss in the ornamental fish industry accounting for an average economic loss of 400 million USD [7, 8]. Especially, it results in high mortality rate and loss of attractive traits in wild-caught ornamental fishes under captivity. Introduction of wild fish into a captive condition creates oxidative stress which triggers the glucocorticoids and in turn weakens the immune system making it susceptible to disease [9]. Inappropriate and misapplication of antibiotics to counter pathogens in ornamental fish industry worsens the situation leading to the generation of antimicrobial resistance (AMR) in pathogens [10]. While plant metabolites show strong efficacy in countering AMR pathogens due to their antimicrobial property, synergistic action and ability to interfere with quorum sensing making them an ideal substitute for antibiotics in the ornamental fish sector [11].

Carotenoids are a class of pigments found in many plants, algae and some bacteria. These pigments play a pivotal role in the skin pigmentation of the ornamental fishes since they can't synthesise pigments on their own, and only convert and deposit them in the chromatophores of skin upon ingestion. Apart from their role in enhancing skin pigmentation, carotenoids are known for their significant role in growth promotion, stress resistance, antioxidant properties, immune and reproductive functions in fish [12, 13]. These carotenoids are sensitive molecules which are labile on exposure to light, heat and oxygen making their extraction procedure difficult and expensive [14]. This is one of the underlying reasons behind the increased cost of ornamental fish feeds, with carotenoid sources alone contributing to around 15%–20% of feed cost [4].

The existence of diverse problems in ornamental fish sector warrants the requirement of a multi-functional solution. The phytobiotics which employ the use of plant metabolites, is not an uncommon approach in aquaculture. However, addressing holistic issues along with meeting the expensive carotenoid requirements in aquariculture is of prime importance. The metabolites of curry leaf, *Murraya koenigii*, which is a tropical inexpensive shrub or tree with worldwide distribution, can act as a super supplement to resolve the underlying problems in ornamental fish industry. Furthermore, curry leaf is known for their rich carotenoid and flavonoid contents [15, 16] making it a suitable phytobiotic supplement not only for addressing the multi-dimensional but also industry-specific problems like discolouration of skin pigments and patterns.

Drapefin barb, *Oreichthys crenuchoides* was used as a model animal for this study. *O. crenuchoides* is a tiny barb which has an excellent market value and is known for its peculiar reticulate melanin skin patterns [17, 18] making them an ideal animal for evaluating the plant metabolites on the melanin skin patterns. Apart from the regular traits of an ornamental fish, non-chromatic nature of this animal helps to answer the question of whether carotenoid administration has any impact on the skin colour of non-chromatic fish similar to colour enhancement of feathers in non-chromatic aves.

## 2. Material and Methods

### 2.1. Plant Metabolite Extraction

The leaves of *M. koenigii* authenticated as per World Flora Online, were cleaned, shade dried and pulverised into fine powder. The phytometabolites in the leaf powder were extracted by soaking it in ethanol at 40°C for 24 h. Later, it was filtered and the filtrate was processed to remove solvent by loading in a rotary evaporator at 40°C. The ethanolic exact obtained was stored in a moisture-free airtight container and stored at 4°C. The yield of phytobiotics of *M. koenigii* (PMK) was quantified gravimetrically,

 

  $$\begin{array}{c} \text{Yield }\left(\%\right)=\frac{\text{Weight of extract obtained}}{\text{Wet weight of leaves utilized}}\times 100\;. \end{array}$$

Further, the quantity of total carotenoids in the PMK was estimated as per Olsan [19].

 

  $$\begin{array}{c} \text{Total carotenoid content}\left(\frac{µ\text{g}}{\text{g}}\right)=\frac{A\times V\times 10\times 10^{6}}{2500\times W}, \end{array}$$

where *A* is the absorbance of the sample; *V* is the volume of the solvent used; 10 is the dilution factor; 10^6^ is the conversion factor from g to µg; 2500 is the absorption coefficient of carotenoid and *W* is the weight of sample used in g.

### 2.2. Experimental Diet

Five experimental feeds containing 0, 5, 10, 15 and 20 g/kg PMK were formulated with commercial ingredients to obtain an iso-nutritional diet. The feed formula of the formulated diet is given in Table 1. The proximate composition of the feed was crosschecked by determining moisture content, crude protein (CP), ether extract (EE), crude fibre (CF), nitrogen-free extract (NFE) and digestible energy (DE) as per AOAC [20].

### 2.3. Experimental Design

Subadult *O. crenuchoides* were collected from natural waters, disinfected with potassium permanganate, acclimated, and weaned to the formulated diet. After a 2-week acclimation period, fish weighing 300.12 ± 2.54 mg were randomly assigned to five treatment groups: 0, 5, 10, 15, and 20 g/kg, with a stocking density of 20 fish per tank (75 L). The formulated diet was administered twice daily for 60 days at 5% of biomass, and feed consumption was monitored by observing the feeding area.

### 2.4. Sampling Procedure

At the end of the experimental period, the sampled fish were photographed immediately under constant illumination for chromaticity analysis [21] and pattern analysis. The microstructure of skin was captured in a light microscope (Olympus Magnus MLX-B, Magnus Opto Systems Pvt. Ltd., India) for visualising melanin dispersion. Later, fish were dissected to collect liver, intestine and muscle tissues intended for biochemical analysis. The collected tissue was homogenised with chilled sucrose solution (0.25 M) to get 5% homogenate for determining the enzymatic activity. The tissue was homogenised using a homogeniser (IKA Ultra-Turrax T18, IKA, Germany) and centrifuged (C-24 Plus Refrigerated Centrifuge, REMI, India) at 5000 rpm at 4°C for 10 min.

### 2.5. Water Quality Monitoring

The physico-chemical characteristics of water namely, dissolved oxygen (DO), temperature, pH, total hardness, total alkalinity and total ammoniacal nitrogen (TAN) were monitored periodically to ensure safety. DO and temperature were recorded daily with the help of a probe (HI 9146, HANNA Instruments, Singapore). pH was measured using Sigma pH metre (pH Testr 3, Sigma–Aldrich, India) once in a week. Total hardness, total alkalinity and TAN were estimated once in 2 weeks as per APHA [22].

### 2.6. Growth Performance and Feed Utilisation Efficiency

The growth performance was assessed by measuring the final weight (mg), weight gain (WG; mg), percent WG (%), specific growth rate (SGR) and survival was recorded. The feed utilisation efficiency was assessed based on feed conversion ratio (FCR), feed efficiency ratio (FER), protein efficiency ratio (PER) and lipid efficiency ratio (LER). The following formulas were used to estimate the above stated indices.

  $$\begin{array}{c} \text{Weight gain }\left(\text{mg}\right)=\text{ Final weight }\left(\text{mg}\right)-\text{ Initial weight }\left(\text{mg}\right), \end{array}$$

  $$\begin{array}{c} \text{Percentage weight gain }\left(\%\right)=\frac{\text{Final weight }\left(\text{mg}\right)-\text{Initial weight (mg)}}{\text{Initial weight (mg)}}\times 100, \end{array}$$

 

  $$\begin{array}{c} \text{Specific growth rate (SGR) }\left(\%/\text{day}\right)=\frac{\text{Ln final weight}-\text{Ln initial weight}}{\text{Duration of experiment (days)}}\times 100, \end{array}$$

  $$\begin{array}{c} \text{Survival rate }\left(\%\right)=\frac{\text{Number of fish that were alive at the end of experiment}}{\text{Number of fish stocked}}\times 100, \end{array}$$

 

  $$\begin{array}{c} \text{Feed conversion ratio (FCR)}=\frac{\text{Feed intake (mg)}}{\text{Body weight gain (mg)}}, \end{array}$$

 

  $$\begin{array}{c} \text{Feed efficiency ratio (FER)}=\frac{\text{Body weight gain (mg)}}{\text{Feed intake (mg)}}, \end{array}$$

 

  $$\begin{array}{c} \text{Protein efficiency ratio (PER)}=\frac{\text{Body weight gain (mg)}}{\text{Crude protein fed (mg)}}, \end{array}$$

 

  $$\begin{array}{c} \text{Lipid efficiency ratio (LER)}=\frac{\text{Body weight gain (mg)}}{\text{Crude lipid fed (mg)}}. \end{array}$$

### 2.7. Digestive Enzyme Activity

The digestive enzymes namely, protease, lipase and amylase activity in the intestinal tissue extract of *O. crenuchoides* were analysed. Protease activity was estimated by casein digestion method as per Beaudet et al. [23]. Lipase activity was determined titrimetrically by Cherry and Crandell [24] method. Amylase activity was measured using dinitro salicylic acid (DNS) method by Rick and Stegbauer [25], which estimates the activity of glucoamylase and alpha amylase. The absorbance values of protease and amylase activity assay were read in single beam UV–VIS spectrophometer (LI-294/296, Lasany International, India).

### 2.8. Metabolic Enzyme Activity

The metabolic enzyme activity was assayed in the tissue extracts of liver and muscle. Aspartate aminotransferase (AST), alanine aminotransferase (ALT) and alkaline phosphatase (ALP) activities were estimated based on International Federation of Clinical Chemistry (IFCC) method using commercial kits (AST: Erba S.G.O.T/XSYS0016; ALT: Erba S.G.P.T/XSYS0017; ALP: Erba Liquixx-M ALP/XSYS0002, India). The changes in the sample absorbance were recorded using a biochemical analyser (URA Semi-automatic analyser, Medsource Ozone Biomedicals, India).

### 2.9. Hematological Profile

The hematological profile of *O. crenuchoides* namely, RBC, WBC, haemoglobin (HGB), haematocrit value (HCT), mass corpuscular volume (MCV), mass corpuscular haemoglobin (MCH), mean corpuscular haemoglobin volume (MCHV), platelets (PLTs), mean platelet volume (MPV) and plateletcrit (PCT) values were analysed using automated haematology analyser (H360 Haematology analyser, Erba Mannheim, Czech Republic).

### 2.10. Antimicrobial Activity

The antimicrobial activity of the PMK was examined by subjecting PMK in agar-disc diffusion and agar-well diffusion assay. The agar-disc diffusion assay was performed using the 18 h old culture of *Aeromonas hydrophila* Ah-F, *Aeromonas veronii* Av-U, *Aeromonas sobria* As-C, *Aeromonas caviae* Ac-C, *Escherichia coli* ATCC 25922 and *Streptococcus agalactiae* LCR1 grown on Mueller–Hinton broth (MHB) at 30°C. The bacterial lawn was prepared by spreading the respective bacterium using sterile cotton swabs separately on Mueller–Hinton Agar (MHA) (HiMedia, India) to get uniform bacterial growth [26]. The sterile discs (HiMedia, India) of 6 mm diameter were placed onto the seeded MHA and then loaded carefully with 10 μL of sterile PMK and the control (ethanol; negative control) separately. The oxytetracycline disc (30 μg/disc; HiMedia, India) was used as a positive control for each bacterium. The plates were incubated at 30°C for 24 h and observed for the zones of inhibition. The zone of inhibition was measured and recorded in mm. While the soft-agar overlay well-diffusion assay was performed as per Hockett and Baltrus [27] with the same bacterial strains.

### 2.11. Chromaticity Analysis

The chromaticity parameters were analysed on CIE-Lab scale viz, *L*^*∗*^ (+, lightness; −, darkness), *a*^*∗*^ (+, redness; −, greenness) and *b*^*∗*^ (+, blueness; −, yellowness) as described by Vitt et al. [21] using Adobe Photoshop CS4. The chroma (*C*^*∗*^) and hue (*H*^*∗*^) values were calculated from *L*^*∗*^, *a*^*∗*^ and *b*^*∗*^ values using the formula as follows:

  $$\begin{array}{c} C^{*}=\sqrt{{\left(a^{*}\right)}^{2}+\;{\left(b^{*}\right)}^{2}};\;H^{*}=\tan^{-1}\left(b^{*}/a^{*}\right). \end{array}$$

### 2.12. Pattern Analysis

The reticulate patterns in the test animal were determined by measuring the relative pattern area of melanophores using Image J software. It was obtained using the following formula.

 

  $$\begin{array}{c} \text{Relative pattern area }\left(\%\right)=\frac{\text{Area of melanised region}}{\text{Total lateral area}}\times 100. \end{array}$$

### 2.13. Integrated Biomarker Response (IBR) Index

IBR was calculated to aggregate multiple biological markers into a single index that summarises the overall health status of an animal. It simplifies the complex data and facilitates holistic health assessment. The IBR index of *O. crenuchoides* upon administered with PMK was assessed as per Chen et al. [28] and Beliaeff and Burgeot [29] with a slight modification. From a range of physiological and biochemical parameters, datasets representing positive and negative health indicators were classified and sorted out. Data normalisation was performed to bring variables with different units and magnitudes into a common scale so that those become comparable, accurate and efficient. The data normalisation was done for the positive and negative traits using the formulas, respectively, as follows:

  $$\begin{array}{c} S_{\text{p}}=\frac{X-\text{Min}}{\text{Max}-\text{Min}};\;S_{\text{n}}=1-\left(\frac{X-\text{Min}}{\text{Max}-\text{Min}}\right). \end{array}$$

(For the negative traits, the normalised values were inverted to reflect the higher values representing physiological well-being and vice-versa, like positive traits.)

Where, *S*_p_, normalised score of positive traits; *S*_n_, normalised score of negative traits; *X*, raw value of the biomarker; Min, minimum value of the respective biomarker across all treatments and Max, maximum value of the respective biomarker across all treatments.

From the normalised scores of positive and negative traits or biomarkers, the IBR index can be calculated using the formula as follows:

 

  $$\begin{array}{c} \text{IBR}=\sum_{i=1}^{n}\text{Si}, \end{array}$$

where *S*_i_ is the normalised score of all biomarkers.

### 2.14. Statistical Analysis

To ascertain the substantial impact of oral administration of PMK on the growth and physiological well-being of *O. crenuchoides*, a one-way ANOVA was carried out for all the assessed variables. Before ANOVA, Shapira-Wilk Test was used to test the normality of data. ANOVA was followed by Tukey HSD Test used to compare the treatment means. SPSS (Version 27.0) was used for all the statistical analysis. Differences were deemed significant at *p* < 0.05. The data were presented as mean ± SE.

## 3. Results

### 3.1. Physico-Chemical Parameters of Water

The physico-chemical parameters of water remained within the desirable limits throughout the experimental period. Further, the water quality parameters in the treatment tanks (Table 2) showed no significant differences throughout the experiment.

### 3.2. Growth Performance and Feed Utilisation Efficiency

The oral administration of PMK has significant impacts on the growth performance and feed utilisation efficiency of *O. crenuchoides*. The significant differences (*p*  < 0.05) were noted in the WG, percentage WG (PWG), SGR, FCR, PER and LER, which symbolised better growth performance and feed utilisation efficiency in PMK administered groups, and their mean values are provided in Table 3.

### 3.3. Digestive Enzyme Activity

**Figure 1:**
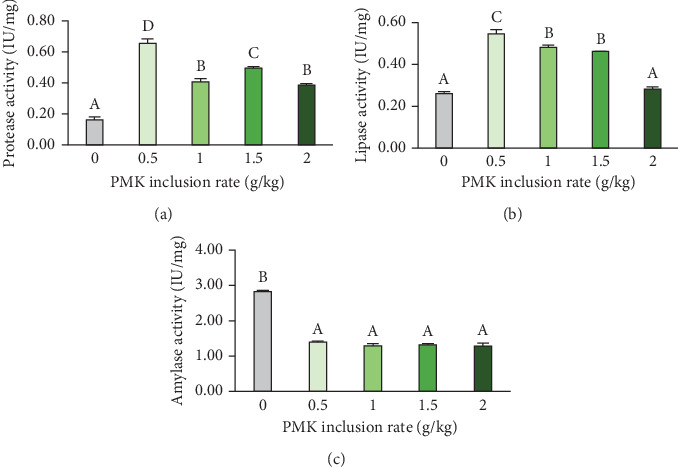
Digestive enzyme activities of *O. crenuchoides* administered with variable ration of PMK. (a) Protease activity, (b) lipase activity and (c) amylase activity. The bars with different capital letters indicate significant differences between them (*p* < 0.001).

The digestive enzyme activity (Figure 1) in the intestinal tissue of *O. crenuchoides* was significantly (*p*  < 0.001) improved by the oral administration of PMK. Notably, protease and lipase activities were found be significantly higher at 5 g/kg (protease: 0.65 ± 0.03 IU/mg; lipase: 0.55 ± 0.02 IU/mg). Amylase, on the other hand, was found to be higher in control fish group (2.82 ± 0.04 IU/mg) and a decreased activity was noted in all PMK administered treatments.

### 3.4. Metabolic Enzyme Activity

**Figure 2:**
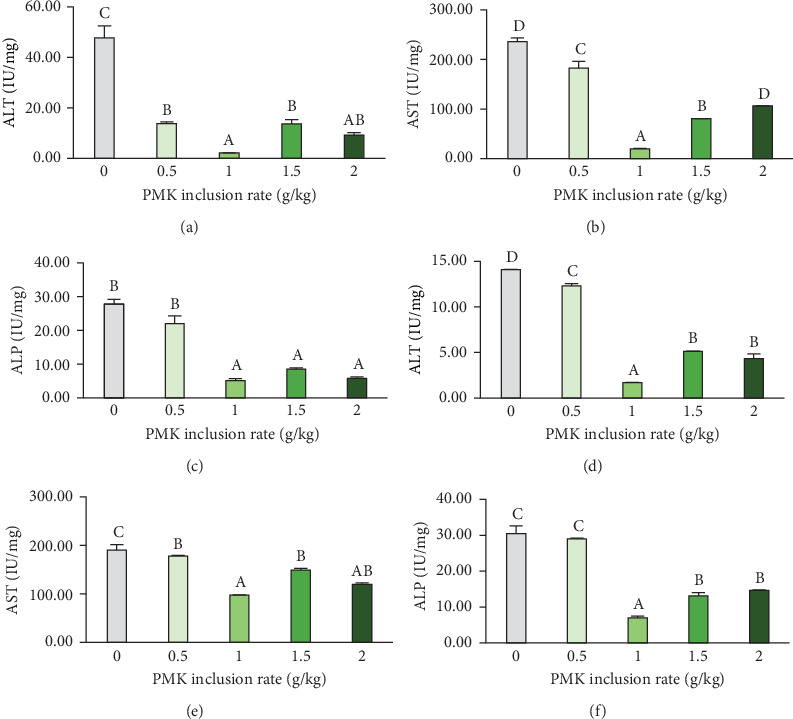
Metabolic enzyme activities in *O. crenuchoides* administered with variable rations of PMK. (a) Liver ALT, (b) liver AST, (c) liver ALP, (d) muscle ALT, (e) muscle AST and (f) muscle ALP activity. The bars with different capital letters indicate significant differences between them (*p* < 0.001).

The metabolic enzyme activity (Figure 2) of *O. crenuchoides* administered with variable rations of PMK was found to have impact in both liver and muscle tissues. In every metabolic enzyme assayed, significantly (*p*  < 0.001) higher enzyme activity was recorded in control fish. Among PMK administered groups, 10 g/kg was noted with the least ALT, AST and ALP activity in both liver and muscle tissues.

### 3.5. Hematological Profile

**Figure 3:**
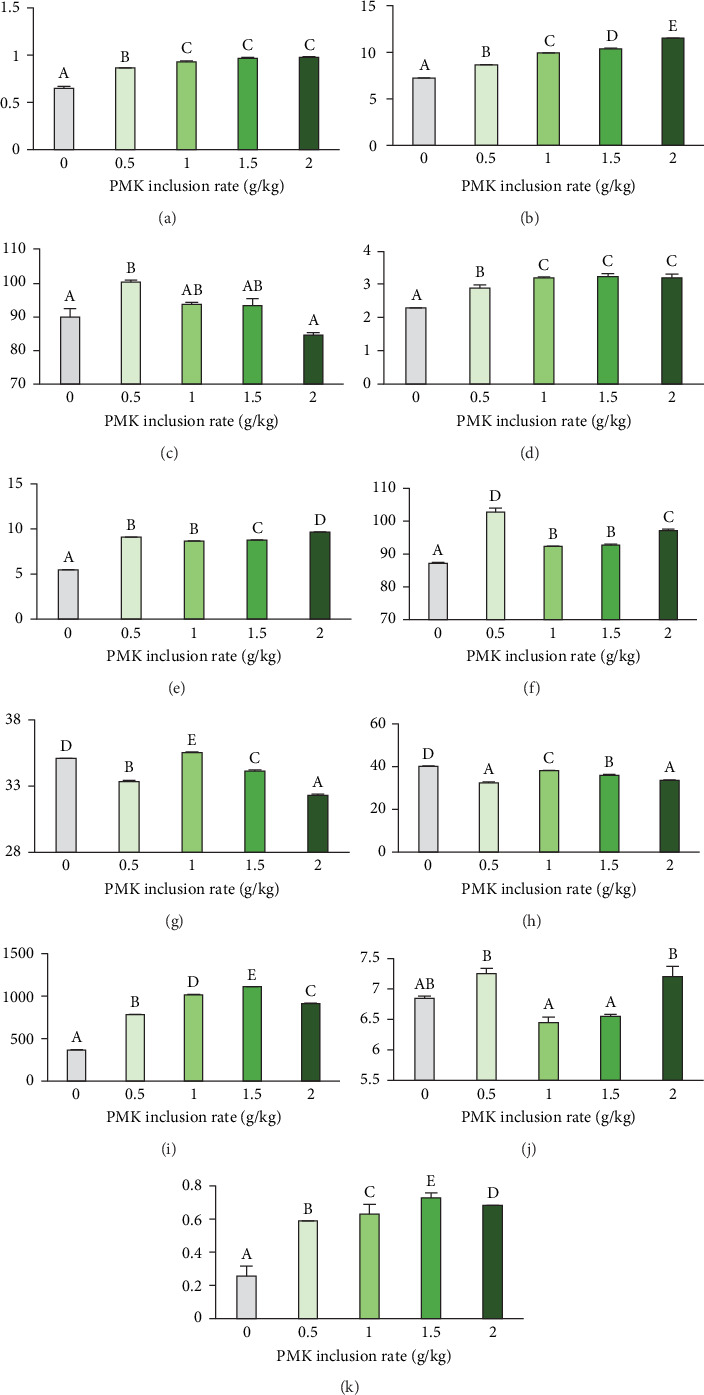
Hematological profile of *O. crenuchoides* administered with PMK. (a) RBC, red blood corpuscles; (b) WBC, white blood corpuscles; (c) RBC/WBC ratio; (d) HGB, haemoglobin; (e) HCT, haematocrit; (f) MCV, mean corpuscular volume; (g) MCH, mean cell haemoglobin; (h) MCHV, mean corpuscular haemoglobin volume; (i) PLT, platelet; (j) MPV, mean platelet volume and (k) PCT, plateletcrit. The bars with different capital letters indicate significant differences between them (*p* < 0.001).

The hematological profile (Figure 3) of *O. crenuchoides* administered with variable rations of PMK exhibited significant differences (*p*  < 0.001) in RBC, WBC, RBC/WBC ratio, PLT, HGB, HCT, MCV, MCH, MCHV, MPV and PCT values. Among various hematological assays, WBC, RBC, HGB and HCT values were significantly higher in fish received 20 g/kg PMK.

### 3.6. Antimicrobial Activity

**Figure 4:**
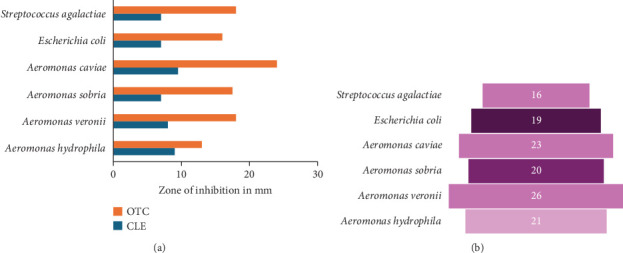
Antimicrobial activity of PMK examined in vitro. (a) Agar-disc diffusion and (b) agar-well diffusion.

The antimicrobial activity (Figure 4) of the PMK examined in vitro showed its positive activity against *Aeromonas hydrophila* Ah-F, *Aeromonas veronii* Av-U, *Aeromonas sobria* As-C, *Aeromonas caviae* Ac-C, *Escherichia coli* ATCC 25922 and *Streptococcus agalactiae* LCR1 in both agar-disc diffusion and agar-well diffusion techniques. The zone of inhibitions from the conducted study provided baseline information on the antimicrobial activity of PMK and can be positively harnessed in combating against the targeted pathogens.

### 3.7. Chromaticity Analysis

**Figure 5:**
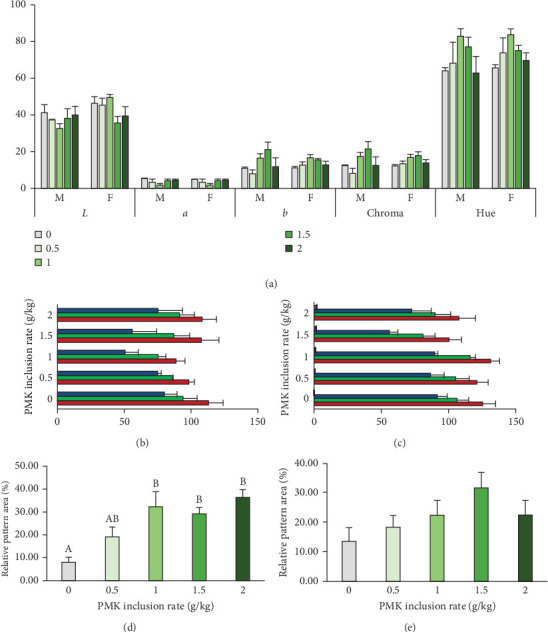
Chromaticity and pattern analysis in the skin of *O. crenuchoides* administered with variable rations of PMK. (a) Chromaticity in CIE-Lab scale of male and female, (b) RGB scale readings of male skin, (c) RGB scale readings of female skin, (d) relative pattern area of male and (e) relative pattern area of female. The bars with different capital letters indicate significant differences between them (*p* < 0.01).

**Figure 6:**
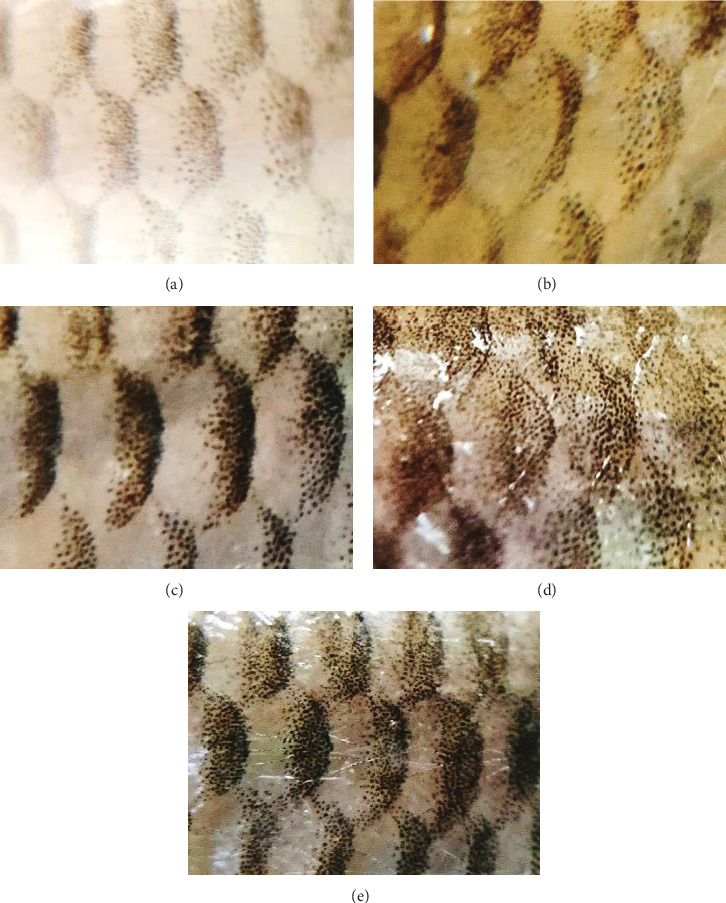
Macroscopic photographs of reticulate skin patterns in *O. crenuchoides* upon variable rations of PMK administration. The reticulate patterns in the skin of *O. crenuchoides* at treatment (a) 0 g/kg, (b) 5 g/kg, (c) 10 g/kg, (d) 15 g/kg and (e) 20 g/kg PMK.

**Figure 7:**
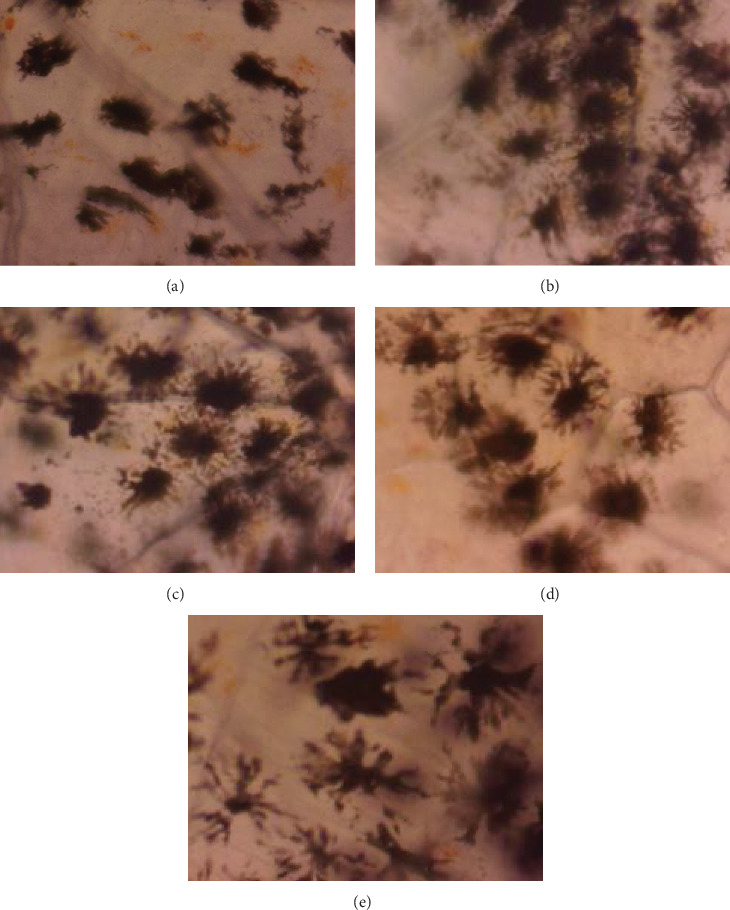
Microscopic photographs of reticulate skin patterns in *O. crenuchoides* upon variable rations of PMK administration. The melanin dispersion in the chromatophores of *O. crenuchoides* at treatment (a) 0 g/kg, (b) 5 g/kg, (c) 10 g/kg, (d) 15 g/kg and (e) 20 g/kg PMK.

Despite the visual light greenish yellow tinge on the chromatophores of the *O. crenuchoides*, the chromaticity analysis on CIE-Lab scale (Figure 5a) showed no significant difference in the *L*^*∗*^, *a*^*∗*^, *b*^*∗*^ and *C*^*∗*^ and *H*^*∗*^ values in both male and female. However, the changes in the CIE-Lab values were in accordance with the visual appearance of *O. crenuchoides*. Briefly, the decreased *L*^*∗*^ and *a*^*∗*^ values in 10 g/kg male represented an improved darkness and greenness in the skin of *O. crenuchoides*, respectively and it was contradictory in female. While the increased *b*^*∗*^, *C*^*∗*^ and *H*^*∗*^ values indicated substantial increase in the yellowness, purity and colour dominance, respectively. Meanwhile, the decrease in all the three channels (R, G and B) in RGB scale also signified the change in hue towards darker shade from paler non chroma (Figure 5b,c). The results of relative pattern area (Figure 5d,e) revealed that it was substantially enlarged in the chromatophores of *O. crenuchoides* due to PMK administration and found to be significant in male (*p*  < 0.01). Figures 6 and 7 show the macroscopic and microscopic views of reticulate patterns in different PMK administered groups.

### 3.8. Correlation Matrix of Parameters Influenced by PMK Administration

**Figure 8:**
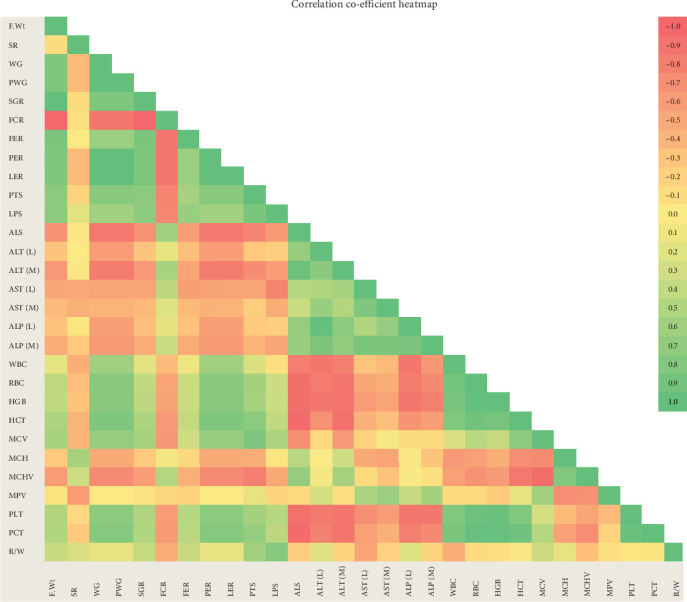
Correlation matrix heatmap depicting the correlation coefficients among various growth and physiological parameters of *O. crenuchoides* on PMK administration. The colour scale indicates correlation co-efficient value ranging from −1 to +1, where vivid green colour represents positive correlation, and vivid red represents negative correlation. ALP (L), liver ALP; ALP (M), muscle ALP; ALS, amylase activity; ALT (L), liver ALT; ALT (M), muscle ALT; AST (L), liver AST; AST (M), muscle AST; FCR, feed conversion ratio; FW, final weight; HCT, haematocrit; HGB, haemoglobin; LER, lipid efficiency ratio; LPS, lipase activity; MCH, mean cell haemoglobin; MCHV, mean corpuscular haemoglobin volume; MCV, mean corpuscular volume; MPV, mean platelet volume; PCT, plateletcrit; PER, protein efficiency ratio; PLS, protease activity; PLT, platelet count; PWG, percentage weight gain; R/W, RBC/WBC ratio; RBC, red blood corpuscles; SGR, specific growth rate; SR, survival rate; WBC, white blood corpuscles; WG, weight gain.

In order to find the relationship between various growth, enzymatic and haematological parameters which were influenced by the administration of PMK in *O. crenuchoides*, correlation coefficient was determined between each parameter and heatmap representing the correlation matrix is shown in the Figure 8. From the heatmap, it was obvious that the digestive enzyme activity was positively correlated with the growth performance of *O. crenuchoides*. While metabolic enzyme activity showed a strong negative correlation with the growth performance and haematological parameters.

### 3.9. IBR Index

**Figure 9:**
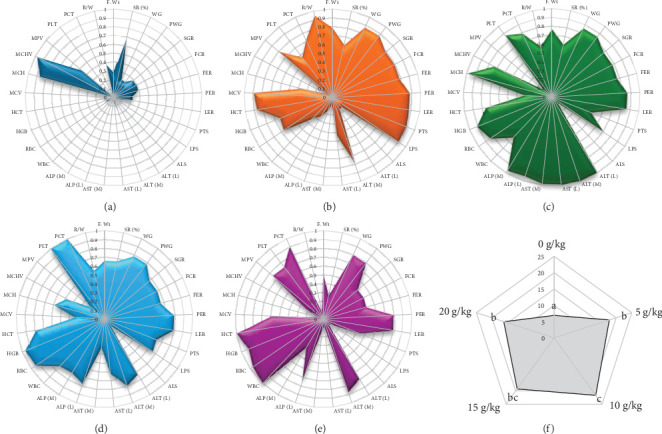
Spider plot representing the performance index (PI) and integrated biomarker response index of *O. crenuchoides* administered with variable rations of PMK. PI of (a) 0 g/kg, (b) 5 g/kg, (c) 10 g/kg, (d) 15 g/kg and (e) 20 g/kg represent normalised value of each parameter and (f) integrated biomarker response index denotes the holistic value considering normalised positive traits and inverted normalised negative traits. ALP (L), liver ALP; ALP (M), muscle ALP; ALS, amylase activity; ALT (L), liver ALT; ALT (M), muscle ALT; AST (L), liver AST; AST (M), muscle AST; FCR, feed conversion ratio; FW, final weight; HCT, haematocrit; HGB, haemoglobin; LER, lipid efficiency ratio; LPS, lipase activity; MCH, mean cell haemoglobin; MCHV, mean corpuscular haemoglobin volume; MCV, mean corpuscular volume; MPV, mean platelet volume; PCT, plateletcrit; PER, protein efficiency ratio; PLS, protease activity; PLT, platelet count; PWG, percentage weight gain; R/W, RBC/WBC ratio; RBC, red blood corpuscles; SGR, specific growth rate; SR, survival rate; WBC, white blood corpuscles; WG, weight gain.

The spider plots of different treatments depicting the respective performance scores are provided in Figure 9. The score of the spider plots corresponds to their performance in each parameter. From the area of spider plots, it can be validated that 10 g/kg dose of PMK had overall better performance followed by 15, 5, 20 and 0 g/kg. Further, IBR index showed a distinct significant difference (*p*  < 0.01) on PMK administration with the highest mean value noted at 10 g/kg (Figure 9).

## 4. Discussion

The current study underscored the impact of oral administration of PMK on the growth and physiological health of *O. crenuchoides* under captivity. PMK administration significantly improved the growth, morpho-chromatic features and biochemical parameters of *O. crenuchoides*, which is evident by the changes in growth performance, feed utilisation efficiency, digestive enzyme activity, metabolic enzyme activity, haematological profile, chromaticity and pattern area analysis. The physico-chemical properties of water used in all treatments for rearing of fish were estimated as the plant metabolites administration in the diet may alter the water quality and microbial community in the aquatic ecosystem and that were found to be within the optimal ranges.

In our investigation, the growth performance and feed utilisation efficiency of *O. crenuchoides* had a marked improvement upon PMK administration. The findings of our study were in accordance with the study carried out by Nuwan et al. [30], who reported that the administration of curry leaf powder enhanced the final WG, average daily gain, FCR and survival of broiler chicks. However, the highest growth performance and feed utilisation were recorded in the lower doses (5 and 10 g/kg) and no dose-dependent dynamics in the growth performance were observed. Previous studies on the metabolic profile of *M. koenigii* have identified several polyphenolic compounds, including kaempferol, quercetin, rutin, gallic acid, chlorogenic acid, catechin and caffeic acid [31], which are well-documented for their potent antioxidant properties and their potential to enhance metabolic efficiency and growth performance. The improved growth performance was mainly due to the efficient feed utilisation mediated by regulated digestive enzyme activity and preserved liver health in PMK-administered groups.

The curry leaf contains essential oils which can potentially stimulate digestive enzymes and enhance their oxidative stability [30]. Proteases are the key enzymes which break down complex proteins in the diet into simple amino acids. The pancreas releases protease into the intestine, where it acts and helps in the assimilation of amino acids which are necessary for protein synthesis [32]. In our study, the highest protease activity was noted at 5 g/kg PMK administration. The increase in protease activity was supported by an in vitro study [33] which confirmed the positive protease activity of the phytoconstituents in curry leaf. Thus, PMK administration can provide exogenous protease support apart from the endogenous protease activity of the animal. This enhanced protease activity facilitated an improved nutrient absorption leading to better growth performance and feed utilisation in the PMK groups. The enzymatic activity of lipase plays a crucial role in breaking and assimilating dietary lipids [34]. The lipase activity assay confirmed better lipase activity at lower doses and a decreasing trend with increasing doses. This phenomenon of any compound which increases the enzymatic activity at lower doses and decreases it at higher doses is known as “hormesis” effect. This occurs due to various reasons, notably their property to act as an enzyme co-factor at lower doses enhancing their catalytic activity and competitive inhibition of the active sites of the substrate at higher doses preventing the enzymes from binding it [35]. The lipid-lowering property of the curry leaf at higher dose may be due to the potential alkaloids which control the activity of pancreatic lipase and restrict lipid absorption [36]. Contradictorily, the enzyme activity of amylase was reduced significantly. This may be due to the presence of novel dimeric carbazole alkaloids [37] having anti-α-amylase and anti-α-glycosidase properties in the PMK [38]. The anti-amylase activity of PMK may be a main reason behind their anti-hyperglycaemic activity which denotes its property in slowing down the release of glucose in the systemic circulation. Additionally, Moon [39] stated that most fish species exhibit intolerance to glucose and Jiang et al. [40] added up that elevated glucose levels increased cortisol levels which is a sign of acute stress. Thus, PMK by controlling the amylase activity regulated the release of glucose in circulation and minimised acute stress due to hyperglycaemia in highfin barb.

Liver is a crucial organ with a key role in growth performance by affecting metabolism, lipid storage and detoxification. Since, administration of any pharmaceutical substance may modulate liver function, assessing the health of liver is of immense importance in ensuring the safety of a diet. The liver function is usually assessed by determining the activity of metabolic enzymes, namely ALT, AST and ALP as they serve as biomarkers of liver health and metabolic syndrome. Any abrupt increase in the activity of these enzymes is often associated with liver damage [41]. ALT and AST are the protein metabolic enzymes which catalyse the transamination of alanine and aspartate to α-ketoglutarate to create pyruvate and oxaloacetate, respectively. Further, these enzymes have their own role in gluconeogenesis. ALP, on the other hand, is involved in the dephosphorylation process which is critical for metabolism and tissue mineralisation [42]. In our study, PMK administration reduced the levels of ALT, AST and ALP with the least mean values identified at 10 g/kg dose. This decrease in the metabolic enzyme activity in PMK-administered groups reflected their nontoxic behaviour upon oral administration and improved liver health. This may be due to the hepatoprotective property of the bioactive compounds present in PMK, indicating its potential in preserving liver health against various contaminants [43, 44].

Apart from the enzymatic biomarkers, haematological profile serves as a blood-related indicator of physiological and metabolic status. In our present investigation, PMK administration caused a significant improvement in the WBC, RBC, HGB, HCT, MCV, MCH, MCHV, PLT, MPV and PCT values of *O. crenuchoides*. HGB is the carrier protein of oxygen packed within every RBC, and its increased level is linked with better aerobic metabolism and higher growth rate. HCT deals with the proportion of blood volume occupied by RBC and its higher values represent a hike in the quantity of RBC and HGB [45]. The PMK administration improved the oxygen carrying capacity of the erythrocytes, which has a direct correlation with the growth rate and metabolic health of an animal. It was evident by the heightened erythrocyte indices, namely MCV, MCH and MCHV that portray the average size of RBC, average amount and concentration of HGB packed within every erythrocyte [46]. These changes facilitate a better oxygen transport and enhance metabolic processes as adequate oxygen is essential for energy production that supports growth. WBC counts are often correlated with the immune health of an animal. In our study, PMK contained immunostimulatory compounds which may be responsible for the elevated WBC counts and triggered immune activity. The increase in RBC, HGB and WBC was in accordance with the findings of Ashry et al. [47]. The elevated PLT count was linked with enhanced clotting and healing abilities. Fish thrombocytes or PLTs are known to possess additional immunological functions apart from clotting and wound healing properties unlike mammalian thrombocytes [48]. Therefore, oral administration of PMK had a substantial positive impact on the haematological parameters, which reflect the growth and immunological well-being of *O. crenuchoides*.

The correlation matrix has given an image of the relationship between various growth and physiological parameters analysed and confirmed the influence of PMK in enhancing the beneficial traits in *O. crenuchoides* by regulating physiological and biochemical parameters. The performance index representation through spider plot portrayed the efficacy of PMK in promoting the growth and physiological parameters. From the spider plots, it can be inferred that 10 g/kg dose resulted in relatively better overall performance. It was evident by the highest area of 10 g/kg in spider plot. The overall effective performance of 10 g/kg dose was confirmed by the significantly highest IBR index value.

AMR in the ornamental fish industry is indeed a growing concern. The ornamental fish industry relies on antibiotics and other antimicrobials to treat diseases in fish. Frequent and inappropriate of antibiotics use can lead to the development of resistance in pathogens and the results are often catastrophic [10]. The inherent compounds of PMK belonging to carbazole alkaloids namely, mahanine, mahanimbine, koenimbine and girinimbine [31] possess antimicrobial activity, which were confirmed by antimicrobial test through agar-disc diffusion and agar-well diffusion assay against selective pathogens. The application of PMK to combat disease and AMR could be effective and safer, unlike conventional antibiotics. The diverse compounds and their synergy have multi-target action which challenges the pathogens to develop resistance [49, 50]. The antimicrobial compounds present in the PMK can eliminate bad gut bacteria and act as a prebiotic for the growth of good bacteria ensuring gut health of the animal. A study by Bhatt et al. [49] proved the antioxidant and prebiotic potential of PMK detailing its efficacy in enhancing the growth of *Lactobacilli sp*. Thus, the bioactive compounds in PMK can potentially suppress the growth of pathogenic bacteria and serve as an alternative to antibiotics in ornamental fish sector.

Vibrant colour and attractive patterns are another important aspect which determines the market price of an ornamental fish. The loss of their colour and patterns may impact their aesthetics and ultimately their market value. Biologically, colours and patterns are a multicomponent intra or interspecific communication in fish [51]. Despite the genetic contribution, these chromatic features are closely related with the nutritional and physiological status of an animal [52]. From nutritional perspective, carotenoids are dietary pigments without which the animal cannot synthesise skin pigments de novo. These pigments are, hence, necessary to be administered in the diet. Once ingested by the animal, these pigments are absorbed, bio-transformed and deposited in various tissues including the skin [12]. Physiologically, stress serves as an important factor which negatively influences the vibrant pigments and patterns in fish [53]. Meanwhile, *O. crenuchoides* which belongs to the genus *Oreichthys* is known for their golden colour tinge on their skin (“Ore”−“aurum” in Latin, meaning gold; “ichthys” in Greek, meaning fish) [54]. Further, they are known for their peculiar reticulate melanin skin patterns. Unlike other pigments, melanin does not require carotenoids for their synthesis. However, any nutritional or physiological interference can alter the natural pigmentation and pattern in *O. crenuchoides*.

We quantified a total carotenoid content of 10 mg per unit gram of PMK. Previous studies put forward that curry leaf has numerous carotenoids namely, lutein and β-carotene [15], which are responsible for pigment synthesis in fishes. A study by Ezhil and Narayanan [55] stated that curry leaf powder administration substantially improved the skin colouration in Kenyi cichlid, *Pseudotropheus lombardoi*. The carotenoids in PMK may have contributed to the restoration of greenish yellow tinge resembling its natural pigmentation in *O. crenuchoides*. Furthermore, PMK contains numerous flavonoids which may potentially enhance the activity of tyrosinase, the key enzyme involved in the production of melanin [56]. Carletti et al. [57] stated that the functions of flavonoids in plant kingdom are analogous to that of melanins in animal kingdom. Flavonoids identified in PMK namely, cyanidin, luteolin, quercetin and fisetin are known for their positive melanogenic property [58]. This may be the reason behind the enlarged melanin reticulate patterns in the skin of *O. crenuchoides*. Thus, the flavonoids can be used as a supplement in boosting melanogenesis which may be effective in fish species with attractive melanin patterns.

## 5. Conclusion

The oral administration of PMK improved the growth performance and feed utilisation efficiency of *O. crenuchoides*. Enzymatically, PMK positively altered the digestive enzyme and metabolic enzyme activities and confirmed the significance in regulating digestion and liver health. The haematological profile detailed the activity of phytobiotic compounds of PMK in aerobic metabolism and immune system. The substantial antimicrobial activity against selected fish pathogens highlighted their potential in countering disease. Despite its carotenoid content, PMK administration failed to significantly enhance the chromaticity in the skin of non-chromatic *O. crenuchoides*. However, the flavonoids in PMK enlarged the reticulate melanin skin patterns and made it more prominent which is an attractive trait of this species. Thus, oral supplementation of 10 g PMK per kg feed is the most effective dose for enhancing the growth performance and physiological status of *O. crenuchoides*. Therefore, PMK with diverse bioactive compounds can be used to combat multifaceted problems in ornamental fish industry with an integrated multi-functional action.

## Figures and Tables

**Table 1:** Feed formula and proximate composition of the experimental diets.

Treatment^a^	0 g/kg	5 g/kg	10 g/kg	15 g/kg	20 g/kg
Ingredients (%)					
Fish meal	28	28	28	28	28
Soy meal	12.5	12.5	12.5	12.5	12.5
MOC	24	24	24	24	24
Maize flour	7.5	7.5	7.5	7.5	7.5
Wheat flour	7.5	7.5	7.5	7.5	7.5
DORB	10	10	10	10	10
Fish oil	4	4	4	4	4
Vitamin-mineral premix	3	3	3	3	3
Vitamin C	0.25	0.25	0.25	0.25	0.25
Betaine HCl	0.05	0.05	0.05	0.05	0.05
Choline chloride	0.2	0.2	0.2	0.2	0.2
CMC	1	1	1	1	1
PMK	0	0.5	1	1.5	2
Dextrin	2	1.5	1	0.5	0
Total	100	100	100	100	100
Average proximate composition (on dry matter basis)					
Moisture (%)	8.31	8.44	8.41	8.41	8.44
CP (%)	35.01	34.97	34.97	34.96	34.97
EE (%)	7.93	7.86	7.86	7.86	7.94
TA (%)	9.16	9.19	8.81	9.22	8.95
CF (%)	4.76	4.89	4.79	4.86	4.89
NFE (%)	34.84	34.75	35.15	34.68	34.84
DE (kcal/100g)	350.77	349.65	351.28	349.34	351.63

*Note:* PMK, curry leaf extract; vitamin-mineral premix, chelated agrimin forte procured from Virbac, India; composition (quantity/kg): vitamin A 7, 00,000 IU; vitamin D3 70,000 IU; vitamin E 250 mg; nicotinamide 1000 mg; cobalt 150 mg; copper 1200 mg; iodine 325 mg; iron 1500 mg; magnesium 6000 mg; manganese 1500 mg; potassium 100 mg; sodium 5.9 mg; sulphur 7.2g; zinc 9600 mg; calcium 250g; phosphorus 120.7.5g. Moisture, moisture content. DE (Kcal/100 g) = (EE% × 9) + (%CP × 4) + (%NFE × 4).

Abbreviatons: CF, crude fibre; CMC, carboxymethyl cellulose; CP, crude protein; DE, digestible energy; DORB, de-oiled rice bran; EE, ether extract; MOC, mustard oil cake; NFE, nitrogen free extract; TA, total ash.

^a^Treatments, PMK at variable rations.

**Table 2:** Physico-chemical characteristics of water in the experimental treatments.

Treatments^a^	0 g/kg	5 g/kg	10 g/kg	15 g/kg	20 g/kg	*p*-Value
DO^b^	7.13 ± 0.07	7.03 ± 0.09	7.1 ± 0.15	7.13 ± 0.15	7.07 ± 0.18	0.977
pH^c^	7.93 ± 0.03	7.87 ± 0.03	8 ± 0.06	7.8 ± 0.06	8 ± 0.06	0.067
Temperature^d^	28.43 ± 1.13	28.43 ± 1.05	28.33 ± 1.07	28.4 ± 0.98	28.57 ± 1.07	0.918
TA^e^	209.33 ± 4.81	206.67 ± 2.40	205 ± 3.00	209 ± 3.79	202.33 ± 3.28	0.626
TH^f^	190.33 ± 5.36	188 ± 5.69	179.67 ± 4.10	175.33 ± 2.73	185.67 ± 1.45	0.145
TAN^g^	0.03 ± 0.00	0.03 ± 0.01	0.03 ± 0.01	0.03 ± 0.01	0.03 ± 0.01	0.24

*Note:* Data are presented as mean ± SE (*n* = 3).

^a^Treatments, PMK at variable rations.

^b^DO, dissolved oxygen (mg/L).

^c^pH.

^d^Temperature (°C).

^e^TA, total alkalinity (mg/L).

^f^TH, total hardness (mg/L).

^g^TAN, total ammoniacal nitrogen (mg/L).

**Table 3:** Growth performance and feed utilisation efficiency of *O. crenuchoides* administered with variable rations of PMK.

Treatments^1^	0 g/kg	5 g/kg	10 g/kg	15 g/kg	20 g/kg	*p*-Value
FW (mg)	383.23 ± 3.57^a^	395.64 ± 2.89^d^	395.25 ± 3.18^d^	392.55 ± 1.8^c^	388.53 ± 1.99^b^	0.046
WG (mg)	80.13 ± 2.92^a^	95.97 ± 1.75^b^	95.52 ± 0.32^b^	93.88 ± 2.74^b^	94.2 ± 1.96^b^	0.002
PWG (%)	26.62 ± 0.97^a^	31.89 ± 0.58^b^	31.73 ± 0.11^b^	31.19 ± 0.91^b^	31.29 ± 0.65^b^	0.002
SGR (%)	0.40 ± 0.02^a^	0.46 ± 0.01^d^	0.45 ± 0.01^cd^	0.44 ± 0.01^c^	0.43 ± 0.01^b^	0.045
Survival (%)	91.67 ± 3.33	90 ± 2.89	90 ± 5.77	91.67 ± 3.33	81.67 ± 3.33	0.382
FCR	2.36 ± 0.02^d^	2.28 ± 0.02^a^	2.28 ± 0.02^a^	2.3 ± 0.01^b^	2.32 ± 0.01^c^	0.049
FER	0.43 ± 0	0.44 ± 0	0.44 ± 0	0.44 ± 0	0.43 ± 0	0.109
PER	0.25 ± 0.01^a^	0.3 ± 0.01^b^	0.3 ± 0^b^	0.3 ± 0.01^b^	0.3 ± 0.01^b^	0.002
LER	1.11 ± 0.04^a^	1.33 ± 0.02^b^	1.32 ± 0^b^	1.3 ± 0.04^b^	1.3 ± 0.03^b^	0.002

*Note:* Data are presented as mean ± SE (*n* = 3). Mean values with different superscripts in the same row differ significantly (*p* < 0.05) (*n* = 3).

Abbreviations: FCR, feed conversion ratio; FER, feed efficiency ratio; FW, final weight; LER, lipid efficiency ratio; PER, protein efficiency ratio; PWG, percentage weight gain; SGR, specific growth rate; WG, weight gain.

^1^Treatments, PMK at variable rations.

## Data Availability

The data that support the findings of this study are available from the corresponding author upon reasonable request.

## References

[1] PMMSY (2020). https://dof.gov.in/sites/default/files/2020-07/Ornamental_fisheries_development_under_PMMSY.pdf.

[2] Biswas S. P., Singh A. K., Das J. N. (2015). Conservation and Management of Ornamental Fish Resources of Northeast India. *Journal of Aquaculture Research and Development*.

[3] Lau C. C., Mohd Nor S. A., Tan M. P. (2023). Pigmentation Enhancement Techniques During Ornamental Fish Production. *Reviews in Fish Biology and Fisheries*.

[4] Ranjan A. (2016). The Importance of Carotenoids in Aquafeeds « Global Aquaculture Advocate the Importance of Carotenoids in Aquafeeds.

[5] Nurlaili Hikmah, Wijaya R. A., Huda H. M. Potential and Problems of Ornamental Fish Farming Development in Depok City (Case Study: Neon Tetra, Cardinal and Red Nose Ornamental Fish Farmer in Bojongsari District).

[6] Pargunan D., Alagappan M. (2020). Determining and Modelling Consumers’ Preferences for Ornamental Fish Keeping. *International Journal of Farm Sciences*.

[7] Hoseinifar S. H., Maradonna F., Faheem M. (2023). Sustainable Ornamental Fish Aquaculture: The Implication of Microbial Feed Additives. *Animals*.

[8] Evan Y., Putri N. E. Status of Aquatic Animal Health in Indonesia.

[9] Fischer C. P., Romero L. M. (2019). Chronic Captivity Stress in Wild Animals Is Highly Species-Specific. *Conservation Physiology*.

[10] Narendrakumar L., Geetha Preena P., Swaminathan T. R. (2023). Antimicrobial Resistance in Ornamental Fisheries: Causes and Preventive Measures. *Handbook on Antimicrobial Resistance: Current Status, Trends in Detection and Mitigation Measures*.

[11] Keita K., Darkoh C., Okafor F. (2022). Secondary Plant Metabolites as Potent Drug Candidates Against Antimicrobial-Resistant Pathogens. *SN Applied Sciences*.

[12] Haque R., Sawant P. B., Sardar P. (2021). Synergistic Utilization of Shrimp Shell Waste-Derived Natural Astaxanthin with Its Commercial Variant Boosts Physio Metabolic Responses and Enhances Colouration in Discus (*Symphysodon aequifasciatus*). *Environmental Nanotechnology, Monitoring and Management*.

[13] Maoka T. (2020). Carotenoids as Natural Functional Pigments. *Journal of Natural Medicines*.

[14] Kultys E., Kurek M. A. (2022). Green Extraction of Carotenoids From Fruit and Vegetable Byproducts: A Review. *Molecules*.

[15] Shivanna V. B., Subban N. (2012). Carotenoids Retention in Processed Curry Leaves, (*Murraya koenigii* L. Spreng). *International Journal of Food Sciences and Nutrition*.

[16] Singh A. P., Wilson T., Luthria D. (2011). LC-MS-MS Characterisation of Curry Leaf Flavonols and Antioxidant Activity. *Food Chemistry*.

[17] Biswas G., Marbaniang B. (2022). Highfin Barb (*Oreichthys crenuchoides*): A Native Ornamental Fish From Bengal Waters With a Promising Market. *Agriculture and Food E-Newsletter*.

[18] Schäfer F. (2009). *Oreichthys crenuchoides*, a New Cyprinid Fish From West Bengal, India. *Ichthyological Exploration of Freshwaters*.

[19] Olson J. A. (1979). A Simple Dual Assay for Vitamin A and Carotenoids in Human Liver.

[20] AOAC (2006). Official Methods of Analysis. *Association of Official Analytical Chemists*.

[21] Vitt S., Bakowski C. E., Thünken T. (2022). Sex-Specific Effects of Inbreeding on Body Colouration and Physiological Colour Change in the Cichlid Fish *Pelvicachromis taeniatus*. *BMC Ecology and Evolution*.

[22] APHA, Clesceri L. S., Greenberg A. E., Eaton A. D. (2005). *Standard Methods for the Examination of Water and Wastewater*.

[23] Beaudet R., Saheb S. A., Drapeau G. R. (1974). Structural Heterogeneity of the Protease Isolated From Several Strains of *Staphylococcus aureus*. *Journal of Biological Chemistry*.

[24] Cherry I. S., Crandall Jr. L. A. (1932). The Specificity of Pancreatic Lipase: Its Appearance in the Blood After Pancreatic Injury. *American Journal of Physiology-Legacy Content*.

[25] Rick W., Stegbauer H. P. (1974). *α*-Amylase Measurement of Reducing Groups. *Methods of Enzymatic Analysis*.

[26] Prabuseenivasan S., Jayakumar M., Ignacimuthu S. (2006). In Vitro Antibacterial Activity of Some Plant Essential Oils. *BMC Complementary and Alternative Medicine*.

[27] Hockett K. L., Baltrus D. A. (2017). Use of the Soft-Agar Overlay Technique to Screen for Bacterially Produced Inhibitory Compounds. *Journal of Visualized Experiments*.

[28] Chen C. Z., Li P., Wang W. B., Li Z. H. (2022). Response of Growth Performance, Serum Biochemical Parameters, Antioxidant Capacity, and Digestive Enzyme Activity to Different Feeding Strategies in Common Carp (*Cyprinus carpio*) Under High-Temperature Stress. *Aquaculture*.

[29] Beliaeff B., Burgeot T. (2002). Integrated Biomarker Response: A Useful Tool for Ecological Risk Assessment. *Environmental Toxicology and Chemistry: An International Journal*.

[30] Nuwan K. S., Wickramasuriya S. S., Jayasena D. D. (2016). Evaluation of Growth Performance, Meat Quality and Sensory Attributes of the Broiler Fed a Diet Administered With Curry Leaves (*Murraya koenigii*). *Korean Journal of Poultry Science*.

[31] Mondal P., Natesh J., Penta D., Meeran S. M. (2022). Extract of *Murraya koenigii* Selectively Causes Genomic Instability by Altering Redox-Status via Targeting PI3K/AKT/Nrf2/Caspase-3 Signaling Pathway in Human Non-Small Cell Lung Cancer. *Phytomedicine*.

[32] López-Otín C., Bond J. S. (2008). Proteases: Multifunctional Enzymes in Life and Disease. *Journal of Biological Chemistry*.

[33] Sharmila S., Rebecca L. J., Das M. P., Saduzzaman M. (2012). Isolation and Partial Purification of Protease from Plant Leaves. *Journal of Chemical and Pharmaceutical Research*.

[34] Okada T., Morrissey M. T. (2007). Production of n− 3 Polyunsaturated Fatty Acid Concentrate From Sardine Oil by Lipase-Catalyzed Hydrolysis. *Food Chemistry*.

[35] Mattson M. P. (2008). Hormesis Defined. *Ageing Research Reviews*.

[36] Reynaldi M. I., Santoso S., Tjahjono K. (2021). The Effect of Stratified Doses of Curry Leaf Extract (*Murraya koenigii*) on Total Cholesterol and Triglycerides in Male Sprague-Dawley Rats Induced by High Fat Feed. *Journal of Kedokteran Diponegoro (Diponegoro Medical Journal)*.

[37] Sampath S. N. T. I., Jayasinghe S., Attanayake A. P., Karunaratne V., Yaddehige M. L., Watkins D. L. (2022). A New Dimeric Carbazole Alkaloid From *Murraya koenigii* (L.) Leaves With *α*-Amylase and *α*-Glucosidase Inhibitory Activities. *Phytochemistry Letters*.

[38] Narkhede M. B. (2012). Evaluation of Alpha Amylase Inhibitory Potential of Four Traditional Culinary Leaves. https://www.researchgate.net/publication/286557878.

[39] Moon T. W. (2001). Glucose Intolerance in Teleost Fish: Fact or Fiction?. *Comparative Biochemistry and Physiology Part B: Biochemistry and Molecular Biology*.

[40] Jiang D., Wu Y., Huang D., Ren X., Wang Y. (2017). Effect of Blood Glucose Level on Acute Stress Response of Grass Carp *Ctenopharyngodon idella*. *Fish Physiology and Biochemistry*.

[41] Oliveira J., Oliva-Teles A., Couto A. (2024). Tracking Biomarkers for the Health and Welfare of Aquaculture Fish. *Fishes*.

[42] Samanta P., Pal S., Mukherjee A. K., Ghosh A. R. (2014). Evaluation of Metabolic Enzymes in Response to Excel Mera 71, a Glyphosate-Based Herbicide, and Recovery Pattern in Freshwater Teleostean Fishes. *BioMed Research International*.

[43] Sathaye S., Bagul Y., Gupta S., Kaur H., Redkar R. (2011). Hepatoprotective Effects of Aqueous Leaf Extract and Crude Isolates of *Murraya koenigii* Against In Vitro Ethanol-Induced Hepatotoxicity Model. *Experimental and Toxicologic Pathology*.

[44] Pande M. S., Gupta S. P. B. N., Pathak A. (2009). Hepatoprotective Activity of *Murraya koenigii* Linn Bark. *Journal of Herbal Medicine and Toxicology*.

[45] Esmaeili N. (2021). Blood Performance: A New Formula for Fish Growth and Health. *Biology*.

[46] Sarma P. R., Walker H. K., Hall W. D., Hurst J. W. (1990). Red Cell Indices. *Clinical Methods: The History, Physical, and Laboratory Examinations*.

[47] Ashry A. M., Habiba M. M., Abdel-Warith A.wahab A. (2024). Dietary Effect of Powdered Herbal Seeds on Zootechnical Performance, Hemato-Biochemical Indices, Immunological Status, and Intestinal Microbiota of European Sea Bass (*Dicentrarchus labrax*). *Aquaculture Reports*.

[48] Ortiz M., Esteban M. Á. (2024). Biology and Functions of Fish Thrombocytes: A Review. *Fish and Shellfish Immunology*.

[49] Basu S., Veeraraghavan B., Anbarasu A. (2024). Anti-Bacterial Compounds From Indian Curry-Leaf Tree *Murraya koenigii* Have Potential to Inhibit Carbapenem-Resistant *Streptococcus pneumoniae*. *Clinical Epidemiology and Global Health*.

[50] Ningappa M. B., Dhananjaya B. L., Dinesha R., Harsha R., Srinivas L. (2010). Potent Antibacterial Property of APC Protein From Curry Leaves (*Murraya koenigii* L.). *Food Chemistry*.

[51] Price A. C., Weadick C. J., Shim J., Rodd F. H. (2008). Pigments, Patterns, and Fish Behavior. *Zebrafish*.

[52] Vissio P. G., Darias M. J., di Yorio M. P., Pérez Sirkin D. I., Delgadin T. H. (2021). Fish Skin Pigmentation in Aquaculture: The Influence of Rearing Conditions and Its Neuroendocrine Regulation. *General and Comparative Endocrinology*.

[53] Bhatt S., Singh B., Gupta M. (2020). Antioxidant and Prebiotic Potential of *Murraya koenigii* and *Brassica oleracea* var. Botrytis Leaves as Food Ingredient. *Journal of Agriculture and Food Research*.

[54] Fishbase [online]. https://fishbase.mnhn.fr/summary/Oreichthys-crenuchoides.

[55] Ezhil J., Narayanan M. (2013). Enhancement of Pigmentation in Blue Morph, *Pseudotropheus Lombardoi* Through Feeding Different Carotenoid Sources. *World Journal of Fish and Marine Sciences*.

[56] Netcharoensirisuk P., Abrahamian C., Tang R. (2021). Flavonoids Increase Melanin Production and Reduce Proliferation, Migration and Invasion of Melanoma Cells by Blocking Endolysosomal/Melanosomal TPC2. *Scientific Reports*.

[57] Carletti G., Nervo G., Cattivelli L. (2014). Flavonoids and Melanins: A Common Strategy Across Two Kingdoms. *International Journal of Biological Sciences*.

[58] Liu-Smith F., Meyskens F. L. (2016). Molecular Mechanisms of Flavonoids in Melanin Synthesis and the Potential for the Prevention and Treatment of Melanoma. *Molecular Nutrition and Food Research*.

